# Supplementary material for: What nature separated, and human joined together: About a spontaneous hybridization between two allopatric dogwood species (Cornus controversa and C. alternifolia)
Source: PLoS One. 2019 Dec 23;14(12):e0226985. doi: 10.1371/journal.pone.0226985 (PMC6927628; doi:10.1371/journal.pone.0226985)
Supplement: S1 Fig — The compilation based on Thompson et al. (1999) and POWO (2019). (PDF) [file pone.0226985.s001.pdf]

Supporting information

Title: What nature had separated, and human has joined together: about a spontaneous hybridization between two allopatric dogwood species (*Cornus controversa* and *C. alternifolia*)

Authors: Barbara Gawrońska[1*], Maria Morozowska[2], Katarzyna Nuc[1], Piotr Kosiński[2,3], Ryszard Słomski[1]

[1]*Department of Biochemistry and Biotechnology, Faculty of Agronomy and Bioengineering, Poznań University of Life Sciences, Dojazd 11, 60-632 Poznań, Poland.*

[2]*Department of Botany, Faculty of Horticulture and Landscape Architecture, Poznań University of Life Sciences, Wojska Polskiego 7C1, 60-625 Poznań, Poland.*

[3]*Institute of Dendrology, Polish Academy of Sciences, Parkowa 5, 62-035 Kórnik, Poland*

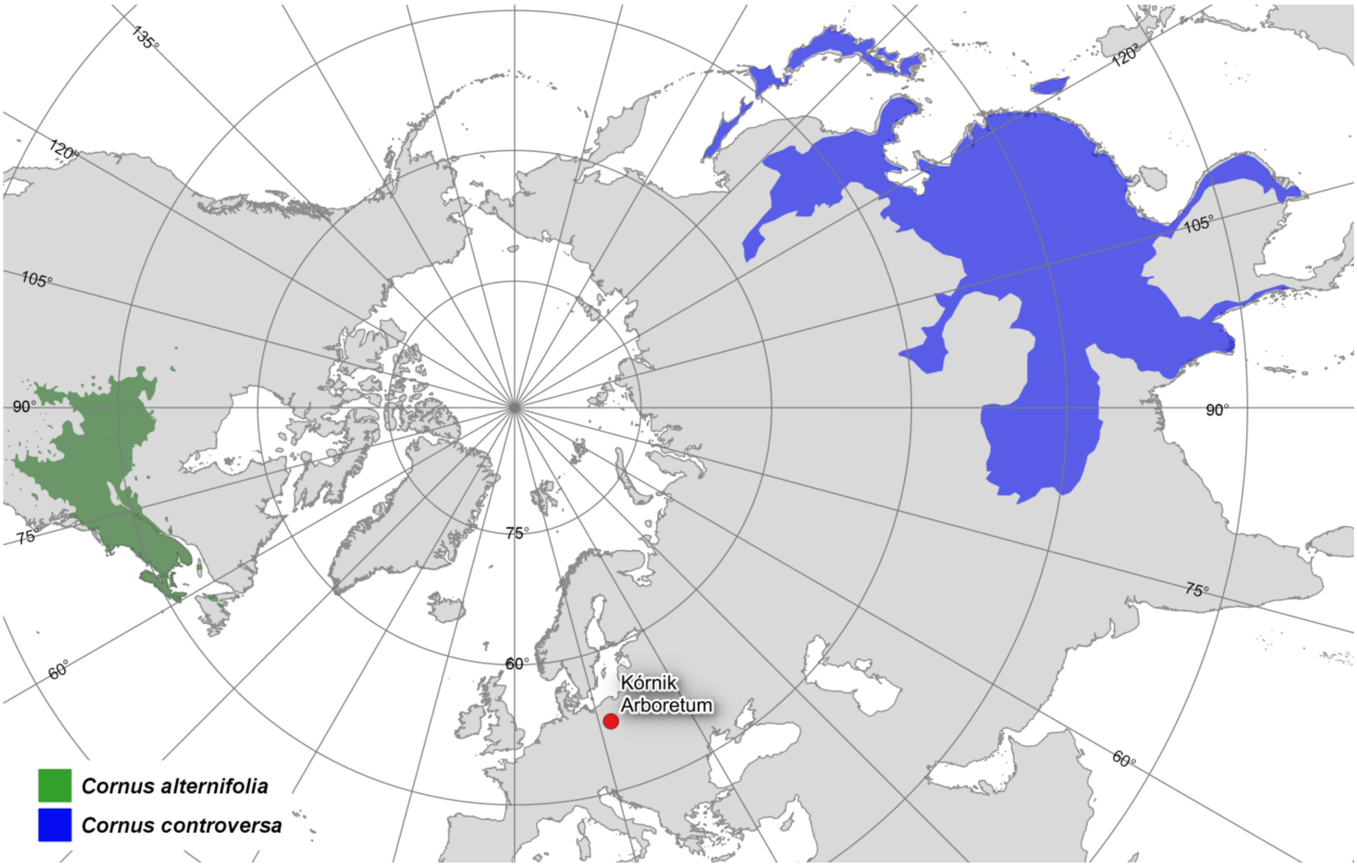

S1 Fig. Geographical distribution of *Cornus alternifolia* and *C. controversa*. The compilation based on Thompson et al. (1999) and POWO (2019)

References

POWO (2019) Plants of the World Online. Facilitated by the Royal Botanic Gardens, Kew. Published on the Internet; http://www.plantsoftheworldonline.org/ (retrieved 02 Aug 2019)

Thompson RS, Anderson KH, Bartlein PJ (1999) Digital representations of tree species range maps from "Atlas of United States trees" by Elbert L. Little, Jr. (1977). In: Atlas of relations between climatic parameters and distributions of important trees and shrubs in North America. Denver, CO: U.S. Geological Survey, Information Services (Producer). On file at: U.S. Department of Agriculture, Forest Service, Rocky Mountain Research Station, Fire Sciences Laboratory, Missoula, MT
