# Supplementary material for: What nature separated, and human joined together: About a spontaneous hybridization between two allopatric dogwood species (Cornus controversa and C. alternifolia)
Source: PLoS One. 2019 Dec 23;14(12):e0226985. doi: 10.1371/journal.pone.0226985 (PMC6927628; doi:10.1371/journal.pone.0226985)
Supplement: S2 Fig — C. alternifolia (a–specimen A9; b–A4; c, d–A2; e–A5; f–A1) and C. controversa (g, h–specimen C21; i–C20; j–C6; k, p–C3; l–C4; m–C5; n–C1, o–C2). Hybrid specimens C1-C5 (k-p), show the reticulate microornamentation pattern with parallel (f, k, m) or wavy (a-e, g, i, j, l, p) cuticle striations, raised (a, c, e, k, l, o, p) or flat (b, d, f, h, j, m, n) anticlinal cell walls and straight pseudo-filiform trichomes (e, f, n, o, p). Magnitude 915–1200; specimen symbols as in Table 1. (PDF) [file pone.0226985.s002.pdf]

Supporting information

Title: What nature had separated, and human has joined together: about a spontaneous hybridization between two allopatric dogwood species (*Cornus controversa* and *C. alternifolia*)

Authors: Barbara Gawrońska[1*], Maria Morozowska[2], Katarzyna Nuc[1], Piotr Kosiński[2,3], Ryszard Słomski[1]

[1]*Department of Biochemistry and Biotechnology, Faculty of Agronomy and Bioengineering, Poznań University of Life Sciences, Dojazd 11, 60-632 Poznań, Poland.*

[2]*Department of Botany, Faculty of Horticulture and Landscape Architecture, Poznań University of Life Sciences, Wojska Polskiego 7C1, 60-625 Poznań, Poland.*

[3]*Institute of Dendrology, Polish Academy of Sciences, Parkowa 5, 62-035 Kórnik, Poland*

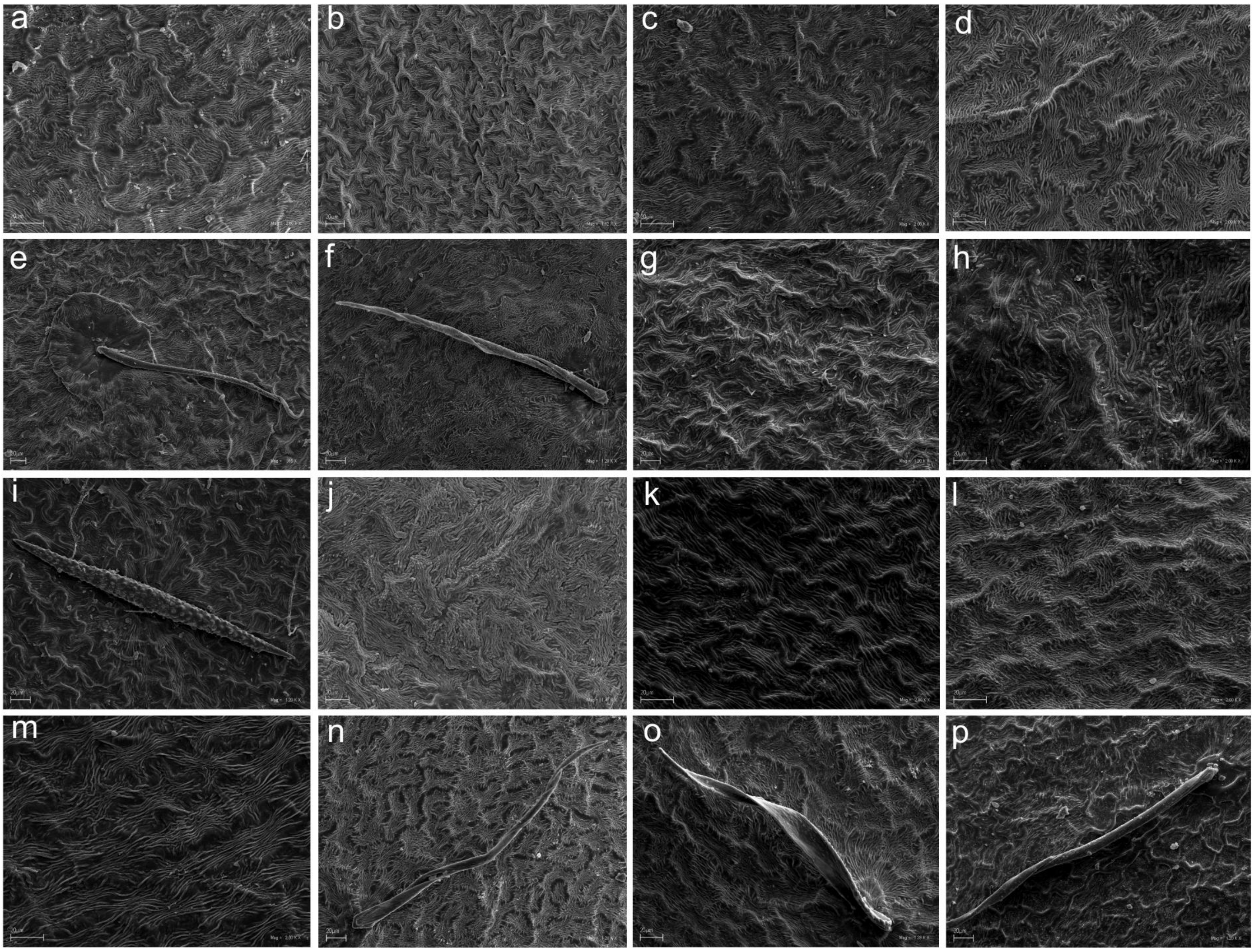

S2 Fig. SEM micrographs of the adaxial leaf surface. *C. alternifolia* (a – specimen A9; b – A4; c, d – A2; e – A5; f – A1) and *C. controversa* (g, h – specimen C21; i – C20; j – C6; k, p – C3; l – C4; m – C5; n – C1, o – C2). Hybrid specimens C1-C5 (k-p), show the reticulate microornamentation pattern with parallel (f, k, m) or wavy (a-e, g, i, j, l, p) cuticle striations, raised (a, c, e, k, l, o, p) or flat (b, d, f, h, j, m, n) anticlinal cell walls and straight pseudo-filiform trichomes (e, f, n, o, p). Magnitude 915-1200; specimen symbols as in Table 1
