# Supplementary material for: What nature separated, and human joined together: About a spontaneous hybridization between two allopatric dogwood species (Cornus controversa and C. alternifolia)
Source: PLoS One. 2019 Dec 23;14(12):e0226985. doi: 10.1371/journal.pone.0226985 (PMC6927628; doi:10.1371/journal.pone.0226985)
Supplement: S4 Fig — Accessions codes as in Table 1; numbers at the nodes indicate bootstrap support. (PDF) [file pone.0226985.s004.pdf]

Supporting information

Title: What nature had separated, and human has joined together: about a spontaneous hybridization between two allopatric dogwood species (*Cornus controversa* and *C. alternifolia*)

Authors: Barbara Gawrońska[1*], Maria Morozowska[2], Katarzyna Nuc[1], Piotr Kosiński[2,3], Ryszard Słomski[1]

[1]*Department of Biochemistry and Biotechnology, Faculty of Agronomy and Bioengineering, Poznań University of Life Sciences, Dojazd 11, 60-632 Poznań, Poland.*

[2]*Department of Botany, Faculty of Horticulture and Landscape Architecture, Poznań University of Life Sciences, Wojska Polskiego 7C1, 60-625 Poznań, Poland.*

[3]*Institute of Dendrology, Polish Academy of Sciences, Parkowa 5, 62-035 Kórnik, Poland*

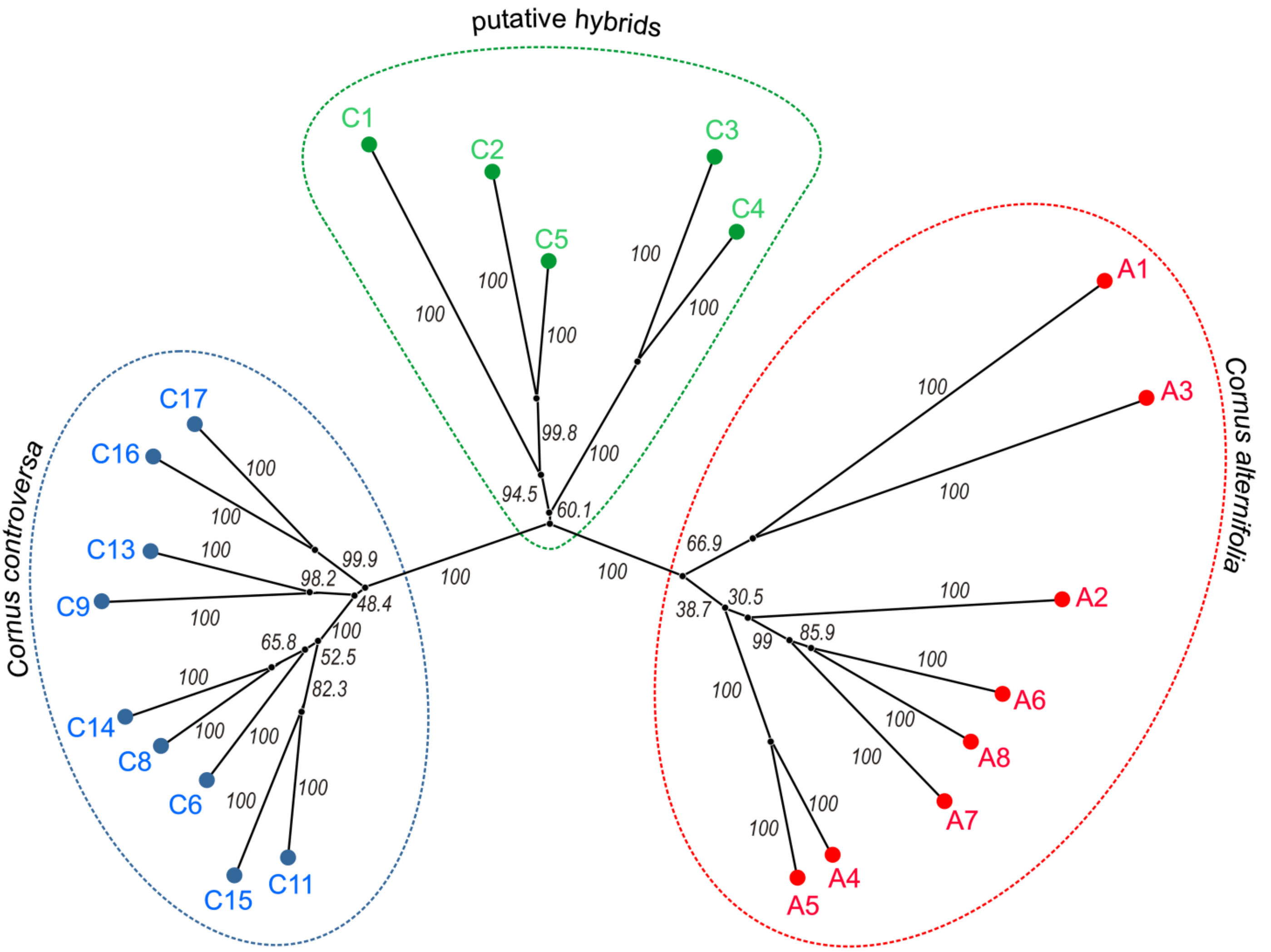

S4 Fig. An unrooted neighbour-joining (NJ) tree of studied *Cornus alternifolia* and *C. controversa* accessions based on Dice distances calculated from RAPD and AFLP combined binary matrices. Accessions codes as in Table 1; numbers at the nodes indicate bootstrap support
