# Supplementary material for: What nature separated, and human joined together: About a spontaneous hybridization between two allopatric dogwood species (Cornus controversa and C. alternifolia)
Source: PLoS One. 2019 Dec 23;14(12):e0226985. doi: 10.1371/journal.pone.0226985 (PMC6927628; doi:10.1371/journal.pone.0226985)
Supplement: S1 Table — The number of DNA bands unique to each parental species and C. macrophylla are compared with the number of species-specific (diagnostic) bands. (PDF) [file pone.0226985.s005.pdf]

Supporting information

Title: What nature had separated, and human has joined together: about a spontaneous hybridization between two allopatric dogwood species (*Cornus controversa* and *C. alternifolia*)

Authors: Barbara Gawrońska[1*], Maria Morozowska[2], Katarzyna Nuc[1], Piotr Kosiński[2,3], Ryszard Słomski[1]

[1]*Department of Biochemistry and Biotechnology, Faculty of Agronomy and Bioengineering, Poznań University of Life Sciences, Dojazd 11, 60-632 Poznań, Poland.*

[2]*Department of Botany, Faculty of Horticulture and Landscape Architecture, Poznań University of Life Sciences, Wojska Polskiego 7C1, 60-625 Poznań, Poland.*

[3]*Institute of Dendrology, Polish Academy of Sciences, Parkowa 5, 62-035 Kórnik, Poland*

S1 Table. Comparison of RAPD and AFLP profiles for *Cornus controversa* (*C.c.*), *Cornus alternifolia* (*C.a.*), putative hybrids (H) and *Cornus macrophylla* (*C.m.*). The number of DNA bands unique to each parental species and *C.macrophylla* are compared with the number of species-specific (diagnostic) bands

Primer/Primer combination	Total products	Polymorphic products	Polymorphism [%]	Bands unique to				Bands species-specific (diagnostic) for	
				C.c.	*C.a.*	H	*C.m.*	*C.c.*	*C.a.*
(A) RAPD									
OPA-1	22	21	95.5	2	7	2	4	2	4
OPA-10	40	40	100	10	3	2	4	1	2
OPA-11	22	22	100	4	7	1	1	0	1
OPA-18	24	21	87.5	4	4	1	2	1	1
OPB-6	26	24	92.3	4	4	2	6	2	1
OPB-12	15	13	86.7	2	1	0	4	0	0
OPB-15	26	26	100	1	5	0	4	0	2
OPB-18	37	35	94.6	4	6	1	3	0	0
OPH-3	24	21	87.5	2	4	2	6	0	3
OPH-5	23	23	100	4	1	0	0	1	2
OPH-7	20	19	95	3	3	2	2	0	1
OPH-8	25	25	100	3	6	2	3	1	4
OPH-13	28	28	100	6	4	3	2	0	0
OPH-18	20	20	100	4	4	1	2	2	0
Total	352	338	96	53	68	19	43	10	21
(B) AFLP									
EcoAAG/MseCAC	205	199	97.1	27	36	14	16	5	11
EcoAAG/MseCAG	200	191	95.5	28	38	13	21	10	6
EcoAAG/MseCTC	233	212	91	8	11	4	28	0	0
EcoAAG/MseCTG	199	182	91.5	26	35	14	27	11	8
EcoACA/MseCTG	163	158	96.9	27	31	7	23	8	5
Total	1000	942	94.2	116	151	52	115	34	30
