# Supplementary material for: What nature separated, and human joined together: About a spontaneous hybridization between two allopatric dogwood species (Cornus controversa and C. alternifolia)
Source: PLoS One. 2019 Dec 23;14(12):e0226985. doi: 10.1371/journal.pone.0226985 (PMC6927628; doi:10.1371/journal.pone.0226985)
Supplement: Attachment — Submitted filename: Response to Reviewers.docx [file pone.0226985.s006.docx]

**Responses to the Editor’s and Reviewers' Comments**

We put forward a corrected version of our manuscript entitled “What nature separated, and human joined together: about a spontaneous hybridization between two allopatric dogwood species (*Cornus controversa* and *C. alternifolia*)”. We would like to thank the Reviewers and the Editor for the effort and time put into the review of the manuscript and for the interest in our work and constructive comments that will greatly improve the manuscript. As indicated below, we have checked all the general and specific comments provided by the Editor and Reviewers. Necessary corrections and supplementations have been introduced to the revised version of our manuscript.

**Reviewer #1 comments:**

1) In the abstract, avoid using acronyms.

*The full names of the used acronyms were introduced into the abstract.*

2) Page 7, section "Plant material", it is not clear to me that how many tree samples are those 30 specimens collected from. If the number is less than 30, then why collecting multiple specimens from same sample?

*Thank you very much for the careful reading of our manuscript. Each sample was taken from a different individual/specimen. As result, we collected nine samples of* C. alternifolia*, 21 samples of* C. controversa *(including putative hybrids), and additionally two samples of* C. macrophylla *(used as an out-group in some analyses). This last piece of information (concerning* C. macrophylla*) was missing in the description of the study material. We added it to the text.*

3) Table 1, what does accession number mean? Does same accession number indicate the same tree/sample?

*As we have mentioned previously, each sample represents different individual. Accession numbers are inventory codes of specimens in given collections. We change caption of the column from ‘Accession number’ to ‘Inventory code’.*

4) It would be interesting if the authors can include samples of the two species from their original places and compare them with the current samples.

*Since both species under study are not native to Europe we tried to do our best in gathering fresh plant material for molecular and micromorphological analyses from specimens either growing in their original places or reproduced from such individuals. The examined by us plant material includes several samples representing specimens of documented natural origin. These are individuals which were reproduced from seeds collected from* C. alternifolia *and* C. controversa *specimens of natural origin (e.g. specimens A2, A5, C9, C16, C17). Additionally, the herbarium material examined under SEM was also obtained from the specimens which collected in natural sites (e.g. specimens A9, C9, C18).*

**Reviewer #2 comments:**

Material
Table 1
The alignment of location and individual is not clear due to some table formatting issues.

*The table layout has been improved.*

A9 location information should be corrected to Fargo, North Dakota.

*Thank you very much for the careful reading of our manuscript. We corrected this flaw.*

Methods
For micromorphological observations, how many samples were used? This is listed for macromorphological analysis. Was it the same for both? If so, then please clarify.

*The micromorphological observations were done on a sample of 5-10 leaves per individual, depending on the material availability. This correction was introduced into the text.*
